# P-850. External Validation of Multiple Bacteremia Prediction Models in a Large US Emergency Department

**DOI:** 10.1093/ofid/ofae631.1042

**Published:** 2025-01-29

**Authors:** Anna G Kaal, Tara Nayak, Laraine Washer, Anastasia Wasylyshyn, Ewout W Steyerberg, Prashant Mahajan, Cees van Nieuwkoop

**Affiliations:** Haga Teaching Hospital, The Hague, Zuid-Holland, Netherlands; University of Michigan Health/Michigan Medicine, Ann Arbor, Michigan; University of MIchigan, Ann Arbor, Michigan; University of Michigan, Ann Arbor, MI; Leiden University Medical Center, Leiden, Zuid-Holland, Netherlands; University of Michigan, Ann Arbor, MI; Haga Teaching Hospital, The Hague, Zuid-Holland, Netherlands

## Abstract

**Background:**

We previously proposed bacteremia prediction models that might save a quarter of blood cultures (BCs) while missing few bacteremia patients. In this study, we aimed to validate these models in the United States.

**Table 1:**
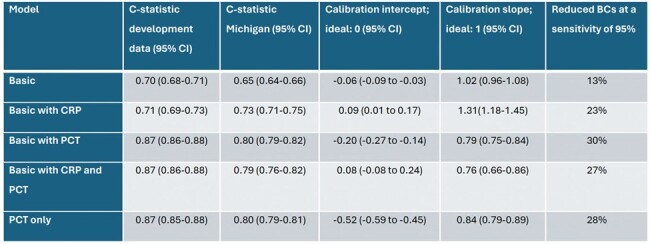
Discrimination, calibration, and clinical usefulness upon external validation for five bacteremia prediction models. Basic variables were age, systolic blood pressure (mmHg), heart rate (beats/min), respiratory rate (breaths/min), temperature (Celsius) and Glasgow coma scale. The basic model was tested in 46236 patients, the model with CRP was tested in 8393 patients, the model with PCT and the only PCT model was tested in 12864 patients and the model with both CRP and PCT was tested in 2317 patients. A sensitivity of 95% means that out of 100 patients with bacteremia, the model detects 95 patients and misses 5 patients. CI = confidence interval, BCs = blood cultures, CRP = C-reactive protein, PCT = Procalcitonin.

**Methods:**

We validated five models using retrospectively collected data. We included patients ≥ 18 years old with one or more BCs drawn during their visit to University of Michigan Hospital, Ann Arbor Emergency Department, from October 2013 until October 2023. The models used basic information such as age and vital signs, with or without the addition of C-reactive protein (CRP), procalcitonin (PCT) or both. We created cohorts based on availability of the biomarkers CRP and PCT. Missing data (except biomarkers) was statistically imputed. Discrimination was assessed with the C-statistic and calibration using calibration plots. Clinical usefulness was evaluated by sensitivity, specificity, and reduced BCs.

**Results:**

We studied 46,608 patients, of whom 372 were excluded because of suspected endocarditis or indwelling vascular catheter. Median age was 61 years, 52% were male. 3816 patients had bacteremia (8.3%), while 2674 (5.8%) patients had contaminated BCs. PCT was known for 12,864 patients, CRP for 8,393 patients and 2,317 patients had both PCT and CRP biomarkers available. We observed substantial improved performance for models containing PCT (Table 1). The best performing model had a C-statistic of 0.80 (95% CI 0.79-0.82), and adequate calibration (intercept -0.20 (95% CI -0.27 to -0.14), slope 0.79 (95% CI 0.75-0.84)). All models somewhat underestimated the risk of bacteremia in the lower risk ranges, leading to more missed bacteremia patients compared to the European cohorts used to develop the models. At a minimum sensitivity of 95%, the models were able to reduce 13-30% of BCs.

**Conclusion:**

Recently proposed prediction models have satisfactory discrimination and calibration for US patients. However, almost all models would have missed more bacteremic episodes compared to the European development cohorts, underlining the importance of validation in other geographical settings. Procalcitonin showed substantial predictive performance for bacteremia and should be considered for any bacteremia prediction model.

**Disclosures:**

**Laraine Washer, MD**, Ferring Pharmaceuticals: Advisor/Consultant

